# Leading for Innovation: Self-Efficacy and Work Engagement as Sequential Mediation Relating Ethical Leadership and Innovative Work Behavior

**DOI:** 10.3390/bs12080266

**Published:** 2022-08-02

**Authors:** Rachadatip Uppathampracha, Guoxin Liu

**Affiliations:** School of Management, Wuhan University of Technology, Wuhan 430070, China; syliuguox@126.com

**Keywords:** ethical leadership, self-efficacy, work engagement, innovative work behavior

## Abstract

This research investigated the link between ethical leadership and innovative work behavior by examining the role of self-efficacy as a mediating factor and the sequential mediation of self-efficacy and work engagement. Using a survey approach, data were collected from 441 bank employees in the southern region of Thailand. The findings of the structural equation modeling (SEM) analysis revealed an association between ethical leadership and innovative work behavior and self-efficacy, respectively. Work engagement and innovative work behavior were both linked to self-efficacy. Work engagement was associated with innovative work behavior. According to the mediation analysis results, self-efficacy appeared to mediate the relationship between ethical leadership and innovative work behavior. Ultimately, it was shown that self-efficacy and work engagement were sequentially mediated by ethical leadership and innovative work behavior. This research provides insight into the understanding of the connection between ethical leadership and innovative work behavior. The key contributions of this research are the exploration-mediating function of self-efficacy and the sequential mediation roles of self-efficacy and work engagement.

## 1. Introduction

Globally, organizations have become more competitive as a consequence of the very unstable organizational environment and intense competition. Innovation is one of the primary drivers of an organization’s competitive advantage [1]. The ability to engage in innovative thinking has become a critical component of organizational success [2]. In this tumultuous climate, the concept that an organization’s existence relies on innovation has been widely accepted [3].

Because of the short product life cycle and globalization, businesses cannot fathom expansion without innovation [4]. In the past, only a few individuals were involved in the quest for innovation; however, now, the whole organization is involved [5]. Their capacity to develop new ideas is essential to the running of their respective firms [6], which makes inventive employees a valued asset [7].

Organizations have adapted to the growing dominating role of employees in the pursuit of innovation [8]. In a similar line, innovative work behavior (IWB) of employees appears to be an essential notion for an organization’s strategic long-term continuity and ambitiousness [2]. Organizations are currently demonstrating an interest in motivating their employees to think of innovative ideas and enact them to enhance their performance. Every innovative idea begins in the thoughts of a person, driving organizations to seek out more creative and inventive employees [9].

Academics have carried out research to find new approaches to encourage employees to engage in IWB [10,11]. They are eager to investigate all antecedents that have the potential to improve employees’ IWB [12]. More study is needed to encourage IWB to successfully harness the innovative potential of employees [13].

Along with the significance of innovation, scholars have recognized the importance of leadership in encouraging and guiding the creative and inventive actions of employees [14]. When it comes to supporting innovation inside their organizations, the role of leadership has multiplied [15]. The search for a leadership style that encourages creativity is one of the topics of study [16,17]. Appropriate leadership has the potential to benefit workers in reaching IWB.

Much additional research has highlighted the effect of diverse leadership styles in achieving greater innovation performance [18,19,20]. Researchers have recently expressed that ethical leadership (EL) encourages IWB [21,22]. Given the growing concern about organizational ethics, it is meaningful to understand how leaders assist their employees in becoming more inventive by implementing ethical practices [23]. Previous research on the association between EL and IWB among employees has been limited [21]. Keeping this in mind, the current study aims to investigate how EL impacts employees’ IWB.

Ethical leaders believe in normatively suitable ethical behaviors and convey these behaviors via their activities. Ethical leaders strive to shift employees’ self-concepts and personal standards to higher objectives and demands [24]. Along these lines, ethical leaders have a major impact on the work attitudes (i.e., self-efficacy) and behaviors (i.e., innovative behavior) of their employees [25]. The association between EL and IWB is based on social exchange theory. Since ethical leaders’ fair and balanced judgments boost employees’ perceptions of being in a social exchange relationship [25,26], employees reciprocate this conduct in the mode of IWB.

When looking at the impact of EL on employees’ innovative conduct, it is important to consider personal aspects [27]. Self-efficacy (SE) is a personality trait that promotes innovation. Individuals with higher SE are more inclined to take on more difficult tasks that require innovative thinking [28]. Hence, if employees perceive SE and expect the outcome of the practice, they will show that behavior to achieve the set goals and are eager to engage in more innovative initiatives for the organization. On the basis of social learning theory, a mediating role for SE in the relationship between EL and employees’ IWB is postulated. This occurs when ethical leaders improve their employees’ SE by setting a good example and talking to them [29].

Moreover, numerous businesses have used work engagement (WE) in terms of collaboration to mobilize employees’ innovative zeal. The reservoir of resources that people require to accomplish their tasks is captured by WE [30]. Some researchers have shown that WE is a positive antecedent of IWB [31,32]. Furthermore, hardly any research has looked at the impact of SE on WE [33]. To fill in these research gaps, this study looked at how SE and WE might act as mediators between EL and IWB.

This study aimed to examine the following research questions:

Research question 1: How does EL impact IWB?

Research question 2: Does SE have a mediating impact on the relationship between EL and IWB?

Research question 3: Do SE and WE have sequentially mediating effects on the relationship between EL and IWB?

This study contributes to social exchange theory and social learning theory by examining how EL is promoted in IWB subordinates through SE and WE based on the empirical study of Thai bank employees. In some other research, alternative mediators, such as psychological empowerment [34], trust [35], perceived meaningful work [22], and self-esteem [21], were employed to examine this relationship. However, no study has specifically investigated the connections between EL and IWB utilizing the above-mentioned mediators. In short, our findings demonstrate how management can be set up to encourage EL among branch managers and SE and WE among employees.

## 2. Theoretical Background

### 2.1. Theoretical Background

Due to leadership being such an important element of the organization, it is likely to affect employee attitudes and behavior significantly. The social exchange theory is a commonly employed theoretical framework for understanding employee attitudes and actions in an organization. It entails a sequence of interactions between individuals [36]. Homans [37] described social exchange as “the exchange of activity, tangible or intangible, and more or less rewarding or costly, between at least two parties”. Furthermore, Blau [38] expressed social exchange by suggesting that the nature of the connection between exchange partners may influence the process of social exchange. It occurs in organizations when specific events lead to interpersonal interactions that are related to social exchange relationships [39]. The supervisor is crucial in the social exchange [40]. Employees who recognize their supervisor’s behavior as encouraging are more likely to engage in more productive activities [41]. Employees prefer to reciprocate with productive actions and attitudes when leaders strive to fulfill them progressively [42].

According to social exchange theory, leaders’ ethical effects on subordinates are reciprocal [43]. Ethical leadership addresses how to manage the organization’s relationship between leaders and employees. In interpersonal interactions, ethical leadership supports employees’ rights and places a premium on equality, freedom, respect, and other essential human rights [44]. As a result of the foregoing discussion, it is possible that employees might realize their own contributions from work; that is, they develop a strong feeling of responsibility for genuine return and are eager to engage in more innovative operations for the business. Similarly, we applied social exchange theory to describe the underlying mechanism relating to ethical leadership and innovative work behavior among employees.

Moreover, the present study also used social learning theory as a theoretical framework to describe the effect of EL on SE [45] and EL with IWB through SE. Bandura [46] explained that human behavior and personality are influenced by three factors: behavior, cognition, and environment. This leads to the notion of SE, which states that a person may assess his or her ability to handle various situations that have occurred to them. According to the general principles of social learning theory, people can learn by seeing others’ behavior, and the effects of that action, and learning, are influenced by behavioral factors.

### 2.2. Conceptual Model

**Figure 1:**
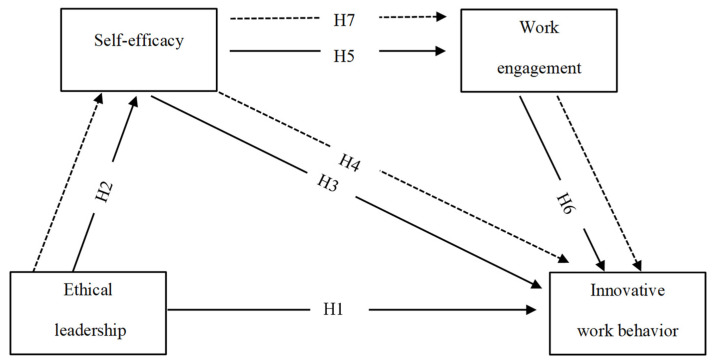
Conceptual model. Note: 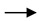 direct effect, 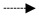 mediating effect.

The principles of social exchange theory suggest that the attitude and behavior of employees are the most strategic and critical aspect of business. Ethical leadership in an organization is the primary determinant of innovative employee work behavior [21]. Figure 1 depicts the conceptual model that we constructed. Obviously, the relationship between EL and IWB is derived from social exchange theory. The social learning theory inspired further direct and indirect paths and the mediated models.

## 3. Hypotheses Development

### 3.1. Ethical Leadership and Innovative Work Behavior

Leadership is one of the essential components of any organization. There are a variety of traits and features that define good leaders [47]. Good leaders are those who are competent and ethical in their actions. Respect, reliability, support, character, altruism, trustworthiness, group motivation, and equity are a few examples of admirable traits [48,49].

Leadership is viewed as a skill that requires theoretical knowledge and the capability to behave ethically and purposefully in certain circumstances based on experience, instinct, discernment, and widespread information [50]. Several business scandals, the global financial crisis, and the recent coronavirus outbreak have all raised awareness of the importance of EL [22]. Academics and practitioners alike respect EL [21]. EL is the promotion of ethical behavior through conscious management and the practice of ethics, in addition to holding all employees accountable for it [51]. Brown, Treviño, and Harrison [24] define EL as the show of strength of appropriate normative behavior through interpersonal relationships and personal actions, combined with Ofori’s [47] endorsement of such suitable conduct to employees through decision-making, support, and two-way correspondence.

In general, EL can be thought of in two ways: individual and management levels. EL is a moral individual who is honest, meritorious, trustworthy, compassionate, and just, and who demonstrates these values in their everyday work and personal life [52,53]. At a management level, ethical leaders who are committed to ethics are supposed to have a positive effect on their employees by promoting integrity, supporting their subordinates, and encouraging their development, and by increasing the significance and independence of work and settling on sensible and reasonable choices [27,54]. Hence, EL is critical in today’s dynamic work environment and an organization’s operation [22].

For the organization to survive and manage a sustainable competitive edge in today’s world, it must constantly innovate to adapt to the external environment [55,56,57]. Employees’ inventive conduct is one of the essential factors in the growth of innovation in the organization and the maintenance of sustainability in an ever-changing world [21]. Additionally, employees’ IWB is important because it is both a requirement for meeting the changing needs of the market today and a source of competitive advantage [58].

IWB refers to a set of behaviors in which employees seek out and discover new opportunities and solutions in the workplace and work to put them into practice, such as actively seeking out and finding new opportunities and arrangements, making arrangements, the generation of ideas, pursuing sponsorship, advancing generated ideas, and conducting feasibility tests [59]. Moreover, introducing some valuable new ideas, processes, or procedures and their implementation is part of an employees’ IWB [60].

Effective leadership behavior is important to encourage employees to engage in IWB. Innovation may be a challenging process loaded with risk [21]. According to this study, EL has an impact on employees’ IWB. It is the result of EL emphasizing the importance of doing work that benefits others. Then, subordinates understand the importance of their work and are enthusiastic about producing advanced ideas and putting them into practice to achieve the organization’s targets [61]. Additionally, EL would consider their subordinates’ jobs dynamic and give them work independence, including the opportunity and judgment to determine their timetable. Thus, they manage their responsibilities and fewer constraints in suggesting, encouraging, and executing creative ideas because more work by self-determination will encourage employees’ IWB [62].

Apart from the previously mentioned study findings, a growing body of evidence points to a link between EL and IWB [22,63]. Considering theoretical arguments and empirical evidence, the researchers may develop the next hypothesis.

**H1.**  *Ethical leadership is positively related to innovativework behavior*.

### 3.2. Ethical Leadership and Self-Efficacy

SE is the most important part of human agency in everyday life, as it turns context knowledge into action [29]. When ethical leaders demonstrate guidance and inspiration [45], employees gain confidence in their competence and reinforce their persuasive and personal conduct standards. This method works towards increasing employees’ SE. By facilitating better SE levels through vicarious or observational learning and seduction, Zhu et al. [64] also demonstrated that kindness and people-oriented ethical leaders enhance employees’ job-related skills, improvement, and confidence. Ethical leaders can also raise followers’ SE by improving expertise and affective arousal through active mastery and attempting to teach employees to ponder their own choices. This mechanism boosts employees’ SE [65].

A psychologically healthy workplace can be generated by ethical leaders who care for their employees and would like them to achieve, where they can engage in active mastery [66]. Employees’ SE rises as a result. Specifically, employees with high SE can outperform others in terms of productivity [45]. Many studies’ findings have backed up the benefit of EL on SE [65,67,68]. As a consequence, the following hypothesis emerged:

**H2.**  *Ethical leadership is positively related to self-efficacy.*

### 3.3. Self-Efficacy and Innovative Work Behavior

SE is described as “people’s beliefs about their capabilities to produce designated levels of performance that exercise influence over events that affect their lives [69].” Individuals who have a strong perception of SE will probably have greater levels of accomplishment and a stronger resolve to withstand irritation and stay fixated when challenges emerge [29]. According to Stajkovic [70], individuals with more certainty are bound to start a new movement, seek them, and keep up with them over the long haul because they are more confident in their ability to manage what they desire to accomplish or what is expected of them. Consequently, employees with higher levels of SE are more likely to come up with, promote, and execute innovative ideas.

IWB is defined as “an individual’s conduct aimed at initiating and intentionally introducing new and valuable ideas, processes, products, or procedures within a work role, group, or organization [71]”. Individuals’ IWB in organization activities include searching for advanced ideas, advocating ideas at work, and collecting funds or setting up to execute ideas [59]. Employees with greater levels of SE are able to participate in more IWB. According to previous research, people with higher levels of SE try to improve work procedures, take on challenging work, and participate in IWB [72,73]. Hence, we proposed the following hypothesis:

**H3.**  *Self-efficacy is positively related to innovative work behavior.*

### 3.4. The Mediating Role of Self-Efficacy

Hypotheses 2 and 3 encourage EL to enhance SE and SE to reinforce IWB, respectively. Ethical leaders influence followers’ SE as appropriate role models who help employees achieve their full capacity at work [29,46]. Furthermore, ethical leaders enhance employees’ IWB by providing opportunities for followers to expand their knowledge. According to social learning theory [29], it is believed that SE plays a mediating function. It is confirmed in the literature that EL is related to IWB via SE as a mediator [73]. This study postulated the following relationship based on the above-mentioned arguments:

**H4.**  *Self-efficacy mediates the relationship between ethical leadership and innovative work behavior.*

### 3.5. Self-Efficacy and Work Engagement

Work engagement is expressed as a pleasant, gratifying, and motivating state of mind marked by vigor, dedication, and absorption concerning their job [30]. High energy levels and mental resilience and a desire to put forth effort and perseverance in the face of adversity describe vigor. A feeling of importance, affection, motivation, pride, and daring are all qualities of dedication. Finally, absorption is defined as complete focus on one’s task [74].

Employees’ SE is essential in increasing their comfort levels when confronted with unexpected occurrences. Shifts in SE are closely linked to shifts in well-being indicators such as engagement [75]. According to Bakker and Demerouti [76], distinct personal active coping styles, such as SE, have been associated with WE. Correspondingly, Pintrich and De Groot [77] stated that SE is a facilitator in the management of cognitive engagement. Additionally, Xanthopoulou et al. [78] noted that the motivating process that leads to engagement is influenced by SE. Along with the previously stated rationale, we hypothesize:

**H5.**  *Self-efficacy is positively related to work engagement.*

### 3.6. Work Engagement and Innovative Work Behavior

Work engagement is an affective–motivational condition of work-related well-being that is permanent and widespread and does not depend on any specific object, situation, or person. [79]. WE has been recognized by academics and practitioners as a factor that should be examined since it may encourage employees to undertake the action [80,81], leading to IWB. Positive employee behaviors, including unrestricted and non-mandatory actions, and ingenuity, creativity, and innovation, are the results of WE [31].

Individuals who are engaged at work are more likely to accept difficult conditions while maintaining their attention and devotion. Because creative work behaviors are difficult to execute owing to the amount of effort required, people’s energy levels, mental resilience, attention, enjoyment, participation, and internal motivation to make a difference aid them in engaging in innovative activities. Engagement at work increases the likelihood that employees will share their work information, effectively communicate new suggestions and new ideas to their colleagues [82], and convert fresh concepts into practical applications. Employees engaged in their work can demonstrate IWB by suggesting and executing ideas that might enhance existing procedures while also opening up new and unexplored opportunities. What follows are some hypotheses based on this evidence:

**H6.**  *Work engagement is positively related to innovativework behavior*.

### 3.7. The Sequential Mediating Role of Self-Efficacy and Work Engagement

Hypotheses 2, 5, and 6 encourage EL to enhance IWB via the mediation of SE and WE. The literature has shown that EL and SE are connected [67]. In addition, multiple studies have shown a link between SE and WE [75] and demonstrated that WE has a positive effect on IWB [31]. According to the findings of Llorens et al. [83], an increase in SE leads to a rise in engagement, which improves the performance, such as IWB.

There is no research investigating the sequential mediating role of SE and WE between EL and IWB. The current study was carried out to address the literature gaps by providing empirical substantiation for the postulated links in the organizational context. Consistent with the upper echelon perspective, we hypothesize:

**H7.**  *Self-efficacy and work engagement sequentially mediate the relationship between ethical leadership and innovative work behavior.*

## 4. Methodology

### 4.1. Overview of Sample and Data Collection

This research utilized data acquired through a survey questionnaire sent across the Thai banking sector. The banking business is characterized by a high reliance on knowledge and expertise, and an abundance of personal contacts [84] due to its highly trained staff, well-established organizational structures, and extensive personal ties.

The banking industry is making ever-increasing contributions to the expansion of the economy [85]. Because banks basically act as mediators between the ultimate borrowers, who are the investors, and the ultimate lending institutions, which are the savers in a community, the growth of the banking industry also has a profound impact on the development of other areas of the economy. This is especially true of the real economy. When it comes to coming up with new products and unique (financial) solutions, the banking industry is one of the most innovative and creative [86].

Employees working in banks in southern Thailand were chosen as respondents because of their expertise in workplace innovation. The research design for this study was based on cross-sectional data collected from bank employees in southern Thailand. In this study, the components were assessed using a self-reported questionnaire. Based on basic random selection, there were 33 banks in operation in Thailand, including 14 commercial banks, 5 state banks, and 15 foreign banks. In this study, only the eight commercial banks with the most branches in the south of Thailand were selected. Data were gathered during November–December 2021, which were operating in the five southern provinces of Thailand.

The participants of this study were picked at random from the employees of the various branches and work units of these banks, with a similar number of responders in each bank. The shortlisted banks’ managers or vice managers were asked to disseminate the questionnaire to their employees. Employees chosen for the survey were informed that their involvement was fully optional and that their identities would be kept private. Employees were asked to evaluate their perception of the branch manager’s ethical leadership in terms of self-efficacy, work engagement, and innovative work behavior. These were self-evaluations.

An overall response rate of 76.03% was achieved by delivering 580 surveys, of which 441 included complete and error-free responses. The participants were asked to fill out a questionnaire that included questions on the factors and some demographic information. The respondents’ profiles are exhibited in Table 1.

### 4.2. Measures

The questionnaires were translated into Thai from English. This study used traditional translation and back-translation procedures to confirm the study instrument’s reliability and validity [87]. This study first translated the original instrument into Thai, then had an English specialist who is Thai translate the Thai version back into English, remarking on any ambiguous elements. Eventually, two local Thai speakers were asked to pre-test the Thai adaptation of the scales, and they tracked down no serious understanding issues.

Ethical leadership was evaluated using a 10-item ethical leadership inventory by Brown, Treviño, and Harrison [24]. Employees replied with 5 points on a Likert, ranging from “1 = strongly disagree” to “5 = strongly agree” to the ethical leadership of their supervisors. The following are some examples of scale items: “My leader defines success not just by results but also the way that they are obtained” and “My leader sets an example of how to do things the right way in terms of ethics.” This is the most extensively employed scale to assess ethical leadership in the literature [88]. The Cronbach’s α value for this scale was found to be 0.96.

Work engagement was examined using 17 items from the scale developed by Schaufeli, Salanova, González-Romá, and Bakker [30]. The scale’s elements indicate three elements: vigor, dedication, and absorption. The vigor and absorption dimensions are made up of six items, but the dedication dimension is made up of five items. In total, the items were appraised on a scale ranging from “1 = never to 7 = always”. The sample items for each dimension are: “I can continue working for very long periods at a time. (vigor),” “I am proud of the work that I do (dedication),” and “I feel happy when I am working intensely (absorption)”. The three elements are highly interconnected, prompting many researchers to suggest calculation of an overall work engagement score for additional analysis [89,90]. The Cronbach’s α value for the scale was 0.96.

Self-efficacy was measured using an 8-item scale by Chen et al. [91]. This scale has been used in several empirical investigations to assess employees’ self-efficacy [92,93]. The sample scale items include “I believe I can succeed at most any endeavor to which I set my mind.” and “I am confident that I can perform effectively on many different tasks”. The responses were assessed on a 5-point scale, ranging from “1 = strongly disagree” to “5 = strongly agree”. The reliability value for the instrument was found to be 0.92.

Innovative work behavior was measured using a 10-item scale by De Jong and Den Hartog [60]. This instrument has been used to evaluate IWB in various empirical studies [31,94]. “I pay attention to issues that are not part of my daily work” and “I attempt to convince people to support an innovative idea” represent items on the sample scale. Employees were polled on their thoughts on the IWB. The items were rated on a 5-point Likert scale from “1 = strongly disagree” to “5 = strongly agree”. The alpha reliability of this instrument was 0.95.

### 4.3. Common Method Variance

All variables this the study were obtained from a single source and at a single point in time. Therefore, common method bias could undermine the validity of the study. We implemented the procedural remedies from [95] to address common methods of variance. We ensured that all necessary permits were obtained and that respondents were adequately informed of the study’s objective and methodology. This was carried out to eliminate response bias among the respondents. In addition, independent and dependent variables were anchored on distinct scales. We did not organize the questionnaire’s constructs according to the examined relationships. Moreover, all scale items were derived from previously established research. Furthermore, we used the statistical solutions proposed by [96]. We conducted a Harman single-factor test to investigate common method bias. The first factor accounted for 38.06 percent of the variance, far less than the 50% criterion [97]. Hence, common method bias was considered to not be a significant issue.

### 4.4. Control Variables

This study consisted of age, gender, education, and organizational tenure, as these criteria have been connected to IWB [98]. Nonetheless, these control factors did not affect IWB. This study anticipated that by including nonsignificant control variables, the degree of freedom would be reduced [99]. Finally, to maintain statistical power, they were excluded from the final analysis.

### 4.5. Data Analysis

With the aid of SPSS PROCESS 24, a regression method was used to analyze the hypotheses, and the effects of mediation were explored using bootstrapping techniques [100]. This approach evaluates direct and indirect effects using simple least square methods. A bootstrapping approach was used to calculate indirect effects.

For the organizational level, this study calculated the interrater agreement (rwg) and intraclass correlations (ICCs) [101]. The between-unit disagreement was measured using the intraclass correlation (ICC) while the within-unit agreement was measured using the inter-rater agreement (rwg) [101,102].

The ICC (1) value should be more than the traditional criteria of 0.05 [102] while the ICC (2) value should be greater than the literature-suggested requirement of 0.70 [103]. The intraclass correlation coefficient (ICC) values from the sample were evaluated to observe whether any consistent variance existed.

The average rwg score was 0.64, with individual rwg values ranging from 0.00 to 0.78, showing that interrater agreement was low [102]. The ICC (1) = 0.06 and ICC (2) = 0.47 were also calculated. There was no requirement for multilevel analysis, and there were no significant differences at the group level to compare [104].

### 4.6. Confirmatory Factor Analyses

The construct validity of the variables was examined before the hypotheses were tested. This study used SPSS AMOS 21 to conduct a series of confirmatory factor analyses (CFA) to explore the uniqueness of the research variables using chi-square statistics and the fit indices of RMSEA, RMR, GFI, NFI, TLI, and CFI [105]. The fit indices supported the four-factor model for EL, SE, WE, and IWB that was proposed: χ2 = 482.990, df = 458, χ^2^/df = 1.055 (*p* = 0.202), RMSEA = 0.011, RMR = 0.015, GFI = 0.943, NFI = 0.969, TLI = 0.998, and CFI = 0.998. The CFA findings confirm the distinctness of the four study variables for analytical purposes even further.

## 5. Results

### 5.1. Measurement Model

This study used the internal consistency, construct convergent validity, and construct discriminant validity to assess the assessment model’s suitability. Cronbach’s alpha (alpha) and composite reliability (CR) checks were applied to define the build dependability. To ensure reliability through interitem consistency, Cronbach’s alpha and CR are normally utilized. Both of them must have a value greater than 0.7 [106,107].

As presented in Table 2, the interitem consistency was established because all the constructions’ alpha and CR values were greater than 0.7. Items must have a factor loading of 0.7 or above [108]. There must be a minimum of 50% variance in the construct for it to be considered valid. There must be a minimum of 0.5 average variance extracted (AVE) for each construct [108]. Convergent validity was determined for both items and constructs, as shown in Table 2, with a reasonable level of loading for each construct exceeding 0.7 and AVE exceeding 0.5, respectively.

The discriminant validity indicated that the model’s constructs were distinct from one another. This research employed the heterotrait–monotrait (HTMT) ratio to determine the discriminant validity. When the HTMT ratio is smaller than 0.9, the discriminant validity is established [109]. Table 2 shows that all the constructs have an HTMT ratio of less than 0.9, indicating discriminant validity. Table 3 presents the factor loading of all constructs. The findings demonstrate that the measurement model is suitable for measuring the constructs in the model.

### 5.2. Structural Model

The postulated construct-to-construct relationships were tested using SPSS PROCESS version 24. The correlational analysis investigated the relationship between the constructs. The correlation between the constructs is shown in Table 4, along with their significant values. IWB was proven to be positively associated with EL, SE, and WE. Similarly, SE was linked to EL and WE favorably. A same conclusion can be drawn about the relationship between WE and EL.

The presence of multicollinearity is evident when the correlation between independent variables exceeds 0.9 [110]. In addition, for independent variables, variance inflation factors (VIFs) are generated to check for multicollinearity. The maximum VIF value that can be used is 4 [107]. The VIF values ranged from 1.027 to 1.076, emphatically demonstrating that multicollinearity was not an issue.

The SPSS PROCESS was utilized in the next structural equation modeling method to discover evidence for the provided hypotheses. Two types of hypotheses were proposed: one looking at the direct effect and the other looking at the mediating influence. It was hypothesized that EL impacts IWB (H1) and SE (H2); SE is positively related to IWB (H3) and WE (H5); and finally, WE is positively related to IWB (H6).

The findings in Table 5 show that EL has a significant impact on employee IWB (β = 0.689, *p* < 0.001). The relationship between EL and SE is significant (β = 0.202, *p* < 0.001). Similarly, SE is also related to IWB (β = 0.087, *p* = 0.014) and SE is associated with WE (β = 0.141, *p* = 0.024). Finally, the relationship between WE and IWB is empirically supported (β = 0.070, *p* = 0.014). Correspondingly, the five direct effect hypotheses are supported.

**Figure 2:**
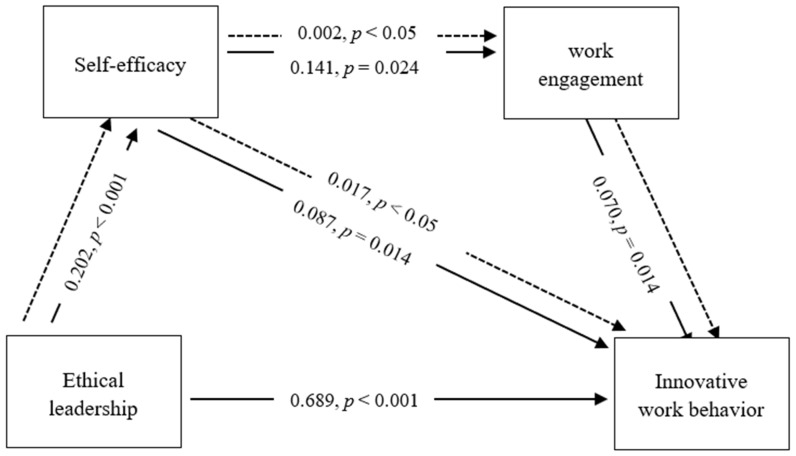
Structural model. Note: 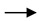 direct effect, 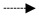 mediating effect.

The final two hypotheses concerned the mediating relationship between EL and IWB. This study identified two mediation relationships: one involving SE and the other involving SE and WE. Both of the connecting pathways were supported by the findings. SE mediated between EL and IWB (β = 0.017, *p* < 0.05). Furthermore, empirical support for the sequential mediating relationship between EL and IWB via SE and WE was discovered (β = 0.002, *p* < 0.05) as displayed in Table 5 (see Figure 2).

Figure 2 depicts the estimated paths. The equation can be expressed as follows: Y3 = a + 0.689X1+ 0.087Y1+ 0.070Y2, when Y3 = IWB, X1 = EL, Y1 = SE, and Y2 = WE. There is no standardized intercept. Specifically, it is equal to zero. As Figure 2 shows, if EL increases for a one SD change, employee IWB will increase by 0.689 SD and employee SE will increase by 0.202 SD. Likewise, if employee SE increases for a one SD change, employee IWB will increase by 0.087 SD and WE will increase by 0.141 SD. In addition, if WE increases for a one SD change, employee IWB will increase by 0.070 SD.

### 5.3. Mediated Hierarchical Regression Analysis

Hierarchical regression was used to improve the precision of the mode, as demonstrated in Table 6. SE was the dependent variable in the first model. Starting with the first stage, EL was revealed to be a significant SE estimator (β = 0.212, *p* < 0.001). In the second model, SE was a significant estimator of WE (β = 0.129, *p* < 0.01) in the first stage.

In the third model, IWB was used as the dependent variable in a three-stage hierarchical multiple regression. In the first stage of the regression, EL was introduced. SE was entered in the second stage, and WE was entered in the third stage. At the outset, EL contributed significantly to the regression model (β = 0.700, *p* < 0.001) and accounted for 49.0% of the variation in IWB according to the hierarchical multiple regression. The addition of SE explained an additional 0.8% of the variation in IWB, with a significant shift in R2 (β = 0.091, *p* <0.01). Finally, the inclusion of WE in the regression model explained an additional 0.7% of the variation in IWB, with a significant change in R2 (β = 0.084, *p* < 0.05). In the third stage of the regression model, all three independent variables were included. EL was the most important predictor of IWB, accounting for 42.9% of the variation in IWB. The three independent variables accounted for 50.5% of the variance in IWB.

## 6. Discussion

This study’s findings support the findings of previous investigations. According to this study’s findings, EL fosters IWB among employees, which is consistent with previous findings [21,22,63]. Because of the various forms of support provided by EL, employees feel important and relevant to the organization, which gives them the confidence to adopt IWB.

This study’s findings indicate that EL is linked to employees’ SE, which is consistent with previous findings linking EL to SE [65,67,68]. Ethical leaders improve the SE of their followers through verbal encouragement and direct examples.

SE has a positive relationship with IWB. These findings align with previous SE research [72,73]. Haines-Gadd [111] also found that training can increase a person’s sense of self-efficacy and that IWB is also linked to certain job-related skills.

SE was found to have a favorable relationship with WE in this study. This study supports a previous finding that showed a link between employee SE and WE [75,78]. The confidence in one’s own competence is a necessity for experiencing vitality and job motivation. Moreover, SE is a prerequisite for commitment since people are more likely to give up if they believe they are utterly incompetent [112].

The last direct link investigated in this study was between WE and employee IWB. The conclusion supports a previous study that found a link between WE and IWB [31,113]. Positive feelings improve thought–action patterns, hence increasing the likelihood of IWB according to the positive effects of WE on IWB.

In this study, two connecting mechanisms between EL and IWB were employed. Two mediating routes were employed in this research: (1) SE as a mediator between EL and IWB; and (2) SE and WE as sequential mediators between EL and IWB of the employees. This study also found that SE plays a role in the relationship between EL and IWB as a partial mediator. This also corresponds to previous literature [73]. This study demonstrates that EL has a beneficial effect on IWB, mostly due to the various sorts of support supplied by EL, which enable employees to have a high level of SE and, as a result, motivates them to engage in IWB. Based on the information available, there is only one piece of evidence indicating the function of SE in mediating the relationship between EL and the employee’s IWB. Furthermore, there are similar pieces of evidence, such as Ma, Cheng, Ribbens, and Zhou [65], which found that the influence of EL on employees’ creativity is mediated by SE. This study contributes to bridging the gap and addressing requirements by introducing self-efficacy mediating variables [27].

As hypothesized, SE and WE sequentially mediate the relationship between EL and IWB. This is one of the first studies to illustrate a sequentially mediated beneficial relationship between EL and IWB. Previous studies revealed the sequential mediating effects of trust and job crafting on the link between servant leadership and IWB [114]. Further, between servant leadership and IWB, psychological empowerment and job crafting were discovered to be sequential mediators [115]. Additionally, psychological empowerment and proactive employee behavior mediate the interaction between transformational leadership and innovative behavior in a sequential approach [116]. This study provides empirical evidence that SE and WE can indeed inspire IWB. Employees who display a high level of SE and WE at work can participate in IWB, paving the way for additional in-depth research links between EL and IWB.

## 7. Theoretical Contribution

This study’s findings provide empirical evidence for the researcher to consider and increase our understanding of EL function in various ways. This study revealed previously undiscovered aspects of the mediating mechanism. This study’s initial contribution is an endeavor to broaden the theoretical lens employed by current research. SE was used as a mediator between EL and IWB in this study. There is only one existing piece of evidence declaring that SE is an appropriate mediator between EL and IWB, as employed by [73].

The social learning theory informed the current study’s SE as a mediator [46]. Leadership guidance entails self-confidence, assertiveness, self-reliance, and the ability to adapt to new conditions, which the supported, facilitated, and appreciated employee utilizes to acquire more SE. Because of its beneficial impacts on employee well-being, SE can boost positive emotions, which employees utilize to generate and implement ideas, resulting in the emergence of second-stage IWB.

This study’s second contribution is the employment of sequential mediators between EL and employee IWB. Employee-centric EL boosts employees’ SE. After, SE enhances WE. Employees eventually adopt IWB as their SE and WE improve. To begin with, they practice SE by altering their capacity to perform the actions needed to accomplish certain performance objectives [46]. Employees’ SE improves their confidence in their abilities and control over their motivation (i.e., work engagement) and behavior (i.e., innovative work behavior). With the increased quantity of energy invested in goal attainment, there is a greater chance of achieving certain levels of behavioral performance [29]. Thereafter, this study indicates that high degrees of WE are associated with high levels of energy, perseverance, identity, and goal-directedness, and high levels of engagement are associated with more proactive work behavior. (i.e., innovative work behavior) [1]. Employees can generate new ideas, which is the first part of IWB. They can also work on putting those ideas into action, which is part of IWB’s implementation.

This empirical evidence from SEM models of the cross-sectional relationships in the sequential mediators model supports the necessity to include a new antecedent and the consequences of IWB. After learning the study’s outcomes, it is proposed that researchers expand the literature by looking at additional different consequences of EL.

## 8. Practical Contribution

These findings have a huge impact on leadership’s contemporary manifestations and framework today, with consequences for benefiting practitioners and managers from this study’s practical recommendations. To begin with, there is a solid correlation between organizational members’ perceptions of EL and their IWB. Therefore, it is suggested that supervisors demonstrate ethical behavior at work by recognizing subordinates’ integrity, inspiring them to offer fresh ideas, and empowering them to execute innovative ideas confidently. Specifically, employees should improve their work by first demonstrating EL, and then starting to display IWB.

Second, as a result of this research, perceived EL favorably influences SE and IWB. Hence, to foster innovation, managers should communicate and exercise their ideas openly with employees. Managers can also use training activities that increase employees’ confidence in their ability to engage in IWB. It has been demonstrated that self-efficacy can be enhanced by training initiatives that emphasize self-management or mastery modeling and supportive supervisory methods, such as delivering positive feedback. They should keep in mind that employees first engage in SE before moving on to IWB. Managers must wait until after they have encouraged SE before introducing or promoting IWB, as IWB emerges once SE has been completely established.

Third, according to this study, SE, which ensures EL, encourages employees to remain in WE, which generates confident emotions, which are then utilized to invest in IWB. To put it another way, employees’ IWB may be fostered by managers’ use of EL to enhance employees’ SE, which in turn can promote WE toward IWB. Managers must be knowledgeable of SE and WE in managing the relationship between the two. The most important practical result of this study is that it suggests that training, development programs, and carrying out directing activities, such as coaching, mentoring, and workshops, can create positive states of mind (i.e., self-efficacy and engagement) for employees in organizations.

Finally, this research provides useful implications for national policy related to the development of banks in Thailand. Fostering EL for bank leaders would directly promote the digital economy of Thailand. The Thai government started its ambitious plan to build Thailand as a digital economy and a regional digital hub in 2016. [117]. In addition to intensive investment, one of the important actions is to boost innovation in this plan through upskilling of employees’ digital skills such as AI and data analytics [117]. The stable development of Thailand is attributed to its bank-based economy. Whether the banking industry can adapt to the competitive innovation environment will determine the development trends of the future digital economy. Meanwhile, our study found that the EL of Thai banks could have an impact on the innovation of the banking industry. Thus, a sufficient number of bank leaders with high EL would contribute to the digital economy of Thailand through innovation from banks.

## 9. Limitations and Future Research

Several limitations in this study suggest additional investigation is needed. To start with, the current study explored the influence of EL on employees’ IWB through the mediating function of SE and WE. More moderating or even mediating variables, such as hope, creativity, and organizational identity, should be included in future studies. Second, in collecting data, future researchers might utilize managers to evaluate themselves by the rate of the EL survey. Managers might also be asked to assess how innovative their employees are at work. Third, less focus was placed on cultural influences when studying the relationship between the various factors examined in this study. Future research could investigate the effect of cultural influences on these variables, as Thailand is a collectivist nation with potentially distinct results from developed countries. Fourth, our research was based solely on individuals. It is advised that collective SE and WE are studied at multiple levels. Therefore, future research must examine how the SE or WE of individual employees contributes to the SE or WE of the entire organization or group. Finally, this study used social exchange theory as the framework for developing hypotheses, which is a novel approach. Building on the tenets of the componential theory of creativity may help to uncover intriguing insights into how an individual’s creativity may contribute to innovative work behavior.

## 10. Conclusions

The aims of this study were to define the impact of EL on encouraging employees to engage in IWB. Additionally, this study aimed to determine how employees’ SE and WE interacted. Furthermore, this study examined the impact of EL on helping employees improve their SE and sought to determine SE’s role in assisting employees in their WE. Moreover, this study also sought to impose the WE in facilitating employees’ IWB. Our findings reflect the premise that certain behavioral psychology, such as self-efficacy and work engagement, is significant in assisting employees to develop innovative work behaviors. We believe that the current results will inspire additional research in this crucial field.

## Figures and Tables

**Table 1:** Respondents’ profile.

Variables	Values	*n* (441)
Gender	Females	75.70%
	Males	24.30%
Education	diploma	1.80%
	Bachelor’s degree	78.30%
	Master’s degree	18.80%
	Ph.D.	1.10%
Age	20–30 years old	35.40%
	31–40 years old	48.60%
	41–50 years old	12.20%
	51–60 years old	3.80%
Organizational tenure	1–10 years	77.20%
	11–20 years	14.70%
	21–30 years	7.00%
	31–40 years	1.10%

**Table 2:** Reliability and validity.

Variables	Min Loading	Alpha	CR	AVE	HTMT Ration		
					(1)	(2)	(3)
1. Ethical leadership	0.843	0.965	0.970	0.764			
2. Self-efficacy	0.812	0.939	0.952	0.767	0.222		
3. Work engagement	0.725	0.946	0.955	0.705	0.113	0.144	
4. Innovative work behavior	0.742	0.956	0.962	0.718	0.730	0.249	0.182

**Table 3:** Factor loadings of constructs.

Latent Variables	Measure Items	Factor Loadings
Ethical leadership	Conducts his/her personal life in an ethical manner	0.872
	Define success not just by results but also the way that they are obtained	0.854
	Listens to what employees have to say	0.864
	Disciplines employees who violate ethical standards	0.887
	Makes fair and balanced decisions	0.891
	Can be trusted	0.889
	Discusses business ethics or values with employees	0.843
	Sets an example of how to do things the right way in terms of ethics	0.900
	Has the best interests of employees in mind	0.849
	When making decisions, asks “what is the right thing to do?”	0.891
Self-efficacy	I will be able to achieve most of the goals that I have set for myself.	0.872
	When facing difficult tasks, I am certain that I will accomplish them.	0.954
	In general, I think that I can obtain outcomes that are important to me.	0.899
	I believe I can succeed at most any endeavor to which I set my mind.	0.917
	I will be able to successfully overcome many challenges.	0.809
	I am confident that I can perform effectively on many different tasks.	0.950
	Compared to other people, I can do most tasks very well.	0.954
	Even when things are tough, I can perform quite well.	0.915
Work engagement	At my work, I feel bursting with energy	0.767
	At my job, I feel strong and vigorous	0.807
	When I get up in the morning, I feel like going to work	0.821
	I can continue working for very long periods at a time	0.831
	At my job, I am very resilient, mentally	0.898
	At my work, I always persevere, even when things do not go well	0.929
	I am enthusiastic about my job	0.882
	I find the work that I do full of meaning and purpose	0.902
	My job inspires me	0.911
	To me, my job is challenging	0.898
	I am proud of the work that I do	0.887
	Time flies when I am working	0.827
	When I am working, I forget everything else around me	0.887
	I feel happy when I am working intensely	0.916
	I am immersed in my work	0.868
	I get carried away when I am working	0.906
	It is difficult to detach myself from my job	0.913
Innovative work behavior	I pay attention to issues that are not part of my daily work.	0.810
	I wonder how things can be improved.	0.743
	I search out new working methods, techniques, or instruments.	0.833
	I generate original solutions for problems.	0.874
	I find new approaches to execute tasks.	0.849
	I make organizational members enthusiastic for innovative ideas.	0.863
	I attempt to convince people to support an innovative idea.	0.852
	I systematically introduce innovative ideas into work practices.	0.885
	I contribute to the implementation of new ideas.	0.868
	I put effort in the development of new things.	0.886

**Table 4:** Means, standard deviations, and correlations.

No.	Variables	Mean	SD	1	2	3	4
1	Ethical leadership	4.50	0.61	1			
2	Self-efficacy	4.37	0.61	0.196 **	1		
3	Work engagement	5.41	0.81	0.110 *	0.127 **	1	
4	Innovative work behavior	4.02	0.69	0.700 **	0.204 **	0.165 **	1

** *p* < 0.01, * *p* < 0.05.

**Table 5:** Structural model.

Effect	Point Estimate	SE	BC 95% CI	
			Lower	Upper
H1: EL→IWB	0.689	0.035	0.619	0.759
H2: EL→SE	0.202	0.045	0.115	0.290
H3: SE→IWB	0.087	0.037	0.014	0.161
H4: SE→WE	0.141	0.062	0.018	0.263
H5: WE→IWB	0.070	0.028	0.014	0.126
H6: EL→SE→IWB	0.018	0.010	0.002	0.041
H7: EL→SE→WE→IWB	0.002	0.001	0.001	0.005

Note(s): EL = ethical leadership, SE = self-efficacy, WE = work engagement, IWB = innovative work behavior.

**Table 6:** Hierarchical regression model.

Stage	Independent Variables	Model 1 Self-Efficacy	Model 2 Work Engagement	Model 3 Innovative Work Behavior
1	Ethical leadership	0.212 ***		0.700 ***
	Self-efficacy		0.129 **	
	R^2^	0.045	0.017	0.490
	ΔR^2^	0.045	0.017	0.490
	ΔF	20.616	7.412	421.259
	Sig. ΔF	0.000	0.007	0.000
2	Ethical leadership			0.681 ***
	Self-efficacy			0.091 **
	R^2^			0.498
	ΔR^2^			0.008
	ΔF			6.867
	Sig. ΔF			0.000
3	Ethical leadership			0.673 ***
	Self-efficacy			0.082 *
	Work engagement			0.084 *
	R^2^			0.504
	ΔR^2^			0.007
	ΔF			0.065
	Sig. ΔF			0.014

*Note.* * *p* < 0.05, ** *p* < 0.01, *** *p* < 0.001.

## Data Availability

The dataset of this study is available from the corresponding author on reasonable request.

## References

[1] Salanova M., Schaufeli W.B. (2008). A cross-national study of work engagement as a mediator between job resources and proactive behaviour. Int. J. Hum. Resour. Manag..

[2] George J.M., Zhou J. (2002). Understanding when bad moods foster creativity and good ones don’t: The role of context and clarity of feelings. J. Appl. Psychol..

[3] Yuan F., Woodman R.W. (2010). Innovative behavior in the workplace: The role of performance and image outcome expectations. Acad. Manag. J..

[4] Lenka U., Gupta M. (2019). An empirical investigation of innovation process in Indian pharmaceutical companies. Eur. J. Innov. Manag..

[5] Mubarik M.S., Govindaraju C., Devadason E.S. (2016). Human capital development for SMEs in Pakistan: Is the “one-size-fits-all” policy adequate?. Int. J. Soc. Econ..

[6] O’Donovan N. (2020). From knowledge economy to automation anxiety: A growth regime in crisis?. New Political Econ..

[7] De Spiegelaere S., Van Gyes G., De Witte H., Niesen W., Van Hootegem G. (2014). On the relation of job insecurity, job autonomy, innovative work behaviour and the mediating effect of work engagement. Creat. Innov. Manag..

[8] Faraz N.A., Mughal M.F., Ahmed F., Raza A., Iqbal M.K. (2019). The impact of servant leadership on employees’ innovative work behaviour-mediating role of psychological empowerment. Int. J. Manag. Sci. Bus. Adm..

[9] Rubera G., Kirca A.H. (2012). Firm innovativeness and its performance outcomes: A meta-analytic review and theoretical integration. J. Mark..

[10] Anser M.K., Yousaf Z., Khan A., Usman M. (2020). Towards innovative work behavior through knowledge management infrastructure capabilities: Mediating role of functional flexibility and knowledge sharing. Eur. J. Innov. Manag..

[11] Khan M.A., Ismail F.B., Hussain A., Alghazali B. (2020). The interplay of leadership styles, innovative work behavior, organizational culture, and organizational citizenship behavior. Sage Open.

[12] Shanker R., Bhanugopan R., Van der Heijden B.I., Farrell M. (2017). Organizational climate for innovation and organizational performance: The mediating effect of innovative work behavior. J. Vocat. Behav..

[13] Agarwal U.A. (2014). Linking justice, trust and innovative work behaviour to work engagement. Pers. Rev..

[14] Friedrich T.L., Mumford M.D., Vessey B., Beeler C.K., Eubanks D.L. (2010). Leading for innovation: Reevaluating leader influences on innovation with regard to innovation type and complexity. Int. Stud. Manag. Organ..

[15] Kim S., Yoon G. (2015). An innovation-driven culture in local government: Do senior manager’s transformational leadership and the climate for creativity matter?. Public Pers. Manag..

[16] Watts L.L., Steele L.M., Den Hartog D.N. (2020). Uncertainty avoidance moderates the relationship between transformational leadership and innovation: A meta-analysis. J. Int. Bus. Stud..

[17] Ali A., Wang H., Soomro M.A., Islam T. (2020). Shared leadership and team creativity: Construction industry perspective. J. Constr. Eng. Manag..

[18] Hoch J.E. (2013). Shared leadership and innovation: The role of vertical leadership and employee integrity. J. Bus. Psychol..

[19] Jiang Y., Chen C.C. (2018). Integrating knowledge activities for team innovation: Effects of transformational leadership. J. Manag..

[20] Zacher H., Rosing K. (2015). Ambidextrous leadership and team innovation. Leadersh. Organ. Dev. J..

[21] Wen Q., Wu Y., Long J. (2021). Influence of ethical leadership on employees’ innovative behavior: The role of organization-based self-esteem and flexible human resource management. Sustainability.

[22] Tahir M. (2020). The role of ethical leadership in promoting employee innovative work behavior mediated by perceived meaningful work among the ICT sector staff in Oman. Int. J. Manag. Entrep. Res..

[23] Tu Y., Lu X., Choi J.N., Guo W. (2019). Ethical leadership and team-level creativity: Mediation of psychological safety climate and moderation of supervisor support for creativity. J. Bus. Ethics.

[24] Brown M.E., Treviño L.K., Harrison D.A. (2005). Ethical leadership: A social learning perspective for construct development and testing. Organ. Behav. Hum. Decis. Process..

[25] Brown M.E., Treviño L.K. (2006). Ethical leadership: A review and future directions. Leadersh. Q..

[26] Mayer D.M., Kuenzi M., Greenbaum R., Bardes M., Salvador R.B. (2009). How low does ethical leadership flow? Test of a trickle-down model. Organ. Behav. Hum. Decis. Process..

[27] Tu Y., Lu X. (2013). How ethical leadership influence employees’ innovative work behavior: A perspective of intrinsic motivation. J. Bus. Ethics.

[28] Carmeli A., Schaubroeck J. (2007). The influence of leaders’ and other referents’ normative expectations on individual involvement in creative work. Leadersh. Q..

[29] Bandura A. (1986). The explanatory and predictive scope of self-efficacy theory. J. Soc. Clin. Psychol..

[30] Schaufeli W.B., Salanova M., González-Romá V., Bakker A.B. (2002). The measurement of engagement and burnout: A two sample confirmatory factor analytic approach. J. Happiness Stud..

[31] Afsar B., Al-Ghazali B.M., Cheema S., Javed F. (2020). Cultural intelligence and innovative work behavior: The role of work engagement and interpersonal trust. Eur. J. Innov. Manag..

[32] Montani F., Vandenberghe C., Khedhaouria A., Courcy F. (2020). Examining the inverted U-shaped relationship between workload and innovative work behavior: The role of work engagement and mindfulness. Hum. Relat..

[33] Chaudhary R., Rangnekar S., Barua M.K. (2012). HRD climate, occupational self-efficacy and work engagement: A study from India. Psychol.-Manag. J..

[34] Ahmad I., Gao Y. (2018). Ethical leadership and work engagement: The roles of psychological empowerment and power distance orientation. Manag. Decis..

[35] Engelbrecht A.S., Heine G., Mahembe B. (2017). Integrity, ethical leadership, trust and work engagement. Leadersh. Organ. Dev. J..

[36] Cropanzano R., Mitchell M.S. (2005). Social exchange theory: An interdisciplinary review. J. Manag..

[37] Homans G.C. (1961). Social Behavior. Its Elementary Forms.

[38] Blau P.M. (2017). Exchange and Power in Social Life.

[39] Cropanzano R., Byrne Z.S., Bobocel D.R., Rupp D.E. (2001). Moral virtues, fairness heuristics, social entities, and other denizens of organizational justice. J. Vocat. Behav..

[40] Iqbal S., Farid T., Ma J., Khattak A., Nurunnabi M. (2018). The impact of authentic leadership on organizational citizenship behaviours and the mediating role of corporate social responsibility in the banking sector of Pakistan. Sustainability.

[41] Anwar A., Abid G., Waqas A. (2019). Authentic leadership and creativity: Moderated meditation model of resilience and hope in the health sector. Eur. J. Investig. Health Psychol. Educ..

[42] Gelens J., Hofmans J., Dries N., Pepermans R. (2014). Talent management and organisational justice: Employee reactions to high potential identification. Hum. Resour. Manag. J..

[43] Zhang Y. (2012). The influence of ethical leadership on employees’ CWB: From social learning and social exchange perspective. J. Bus. Econ..

[44] Xiao G., Zhao Y. (2017). Ethical leadership and employees’ turnover intention: Leader-member exchange as a mediator. Sci. Sci. Manag. ST.

[45] Walumbwa F.O., Mayer D.M., Wang P., Wang H., Workman K., Christensen A.L. (2011). Linking ethical leadership to employee performance: The roles of leader–member exchange, self-efficacy, and organizational identification. Organ. Behav. Hum. Decis. Process..

[46] Bandura A. (1977). Self-efficacy: Toward a unifying theory of behavioral change. Psychol. Rev..

[47] Ofori G. (2009). Ethical leadership: Examining the relationships with full range leadership model, employee outcomes, and organizational culture. J. Bus. Ethics.

[48] Toor S.-U.-R., Ogunlana S.O. (2008). Leadership skills and competencies for cross-cultural construction projects. Int. J. Hum. Resour. Dev. Manag..

[49] Holder M. (2019). Followers say they want leaders with integrity, but do they?. J. Stud. Leadersh..

[50] Kodish S. (2006). The paradoxes of leadership: The contribution of Aristotle. Leadership.

[51] Trevino L.K., Brown M.E. (2004). Managing to be ethical: Debunking five business ethics myths. Acad. Manag. Perspect..

[52] Hansen S.D., Alge B.J., Brown M.E., Jackson C.L., Dunford B.B. (2013). Ethical leadership: Assessing the value of a multifoci social exchange perspective. J. Bus. Ethics.

[53] Lu X., Guy M.E. (2014). How emotional labor and ethical leadership affect job engagement for Chinese public servants. Public Pers. Manag..

[54] Kalshoven K., Den Hartog D.N., De Hoogh A.H. (2011). Ethical leadership at work questionnaire (ELW): Development and validation of a multidimensional measure. Leadersh. Q..

[55] Kuvaas B., Buch R., Dysvik A., Haerem T. (2012). Economic and social leader–member exchange relationships and follower performance. Leadersh. Q..

[56] Tian M., Deng P., Zhang Y., Salmador M.P. (2018). How does culture influence innovation? A systematic literature review. Manag. Decis..

[57] Ren F., Zhang J. (2015). Job stressors, organizational innovation climate, and employees’ innovative behavior. Creat. Res. J..

[58] Abstein A., Heidenreich S., Spieth P. (2014). Innovative work behaviour: The impact of comprehensive HR system perceptions and the role of work–life conflict. Ind. Innov..

[59] Scott S.G., Bruce R.A. (1994). Determinants of innovative behavior: A path model of individual innovation in the workplace. Acad. Manag. J..

[60] De Jong J., Den Hartog D. (2010). Measuring innovative work behaviour. Creat. Innov. Manag..

[61] De Hoogh A.H., Den Hartog D.N. (2008). Ethical and despotic leadership, relationships with leader’s social responsibility, top management team effectiveness and subordinates’ optimism: A multi-method study. Leadersh. Q..

[62] Piccolo R.F., Greenbaum R., Hartog D.N.D., Folger R. (2010). The relationship between ethical leadership and core job characteristics. J. Organ. Behav..

[63] Özsungur F. (2019). The impact of ethical leadership on service innovation behavior: The mediating role of psychological capital. Asia Pac. J. Innov. Entrep..

[64] Zhu W., May D.R., Avolio B.J. (2004). The impact of ethical leadership behavior on employee outcomes: The roles of psychological empowerment and authenticity. J. Leadersh. Organ. Stud..

[65] Ma Y., Cheng W., Ribbens B.A., Zhou J. (2013). Linking ethical leadership to employee creativity: Knowledge sharing and self-efficacy as mediators. Soc. Behav. Personal. Int. J..

[66] Walumbwa F.O., Schaubroeck J. (2009). Leader personality traits and employee voice behavior: Mediating roles of ethical leadership and work group psychological safety. J. Appl. Psychol..

[67] Wang D., Gan C., Wu C., Wang D. (2015). Ethical leadership and employee voice: Employee self-efficacy and self-impact as mediators. Psychol. Rep..

[68] Ren S., Chadee D. (2017). Ethical leadership, self-efficacy and job satisfaction in China: The moderating role of guanxi. Pers. Rev..

[69] Bandura A. (1994). Self-efficacy. Encyclopedia of Human Behavior.

[70] Stajkovic A.D. (2006). Development of a core confidence-higher order construct. J. Appl. Psychol..

[71] Farr J.L., Ford C.M. (1990). Individual innovation. Innovation and Creativity at Work: Psychological and Organizational Strategies.

[72] Hsiao H.-C., Chang J.-C., Tu Y.-L., Chen S.-C. (2011). The impact of self-efficacy on innovative work behavior for teachers. Int. J. Soc. Sci. Humanit..

[73] Zahra T.T., Ahmad H.M., Waheed A. (2017). Impact of Ethical Leadership on Innovative Work Behavior: Mediating Role of Self-Efficacy. J. Behav. Sci..

[74] Salanova M., Llorens S., Cifre E., Martínez I.M., Schaufeli W.B. (2003). Perceived collective efficacy, subjective well-being and task performance among electronic work groups: An experimental study. Small Group Res..

[75] Bresó E., Schaufeli W.B., Salanova M. (2011). Can a self-efficacy-based intervention decrease burnout, increase engagement, and enhance performance? A quasi-experimental study. High. Educ..

[76] Bakker A.B., Demerouti E. (2008). Towards a model of work engagement. Career Dev. Int..

[77] Pintrich P.R., De Groot E.V. (1990). Motivational and self-regulated learning components of classroom academic performance. J. Educ. Psychol..

[78] Xanthopoulou D., Bakker A.B., Demerouti E., Schaufeli W.B. (2007). The role of personal resources in the job demands-resources model. Int. J. Stress Manag..

[79] González-Romá V., Schaufeli W.B., Bakker A.B., Lloret S. (2006). Burnout and work engagement: Independent factors or opposite poles?. J. Vocat. Behav..

[80] Lauring J., Selmer J. (2015). Job engagement and work outcomes in a cognitively demanding context: The case of expatriate academics. Pers. Rev..

[81] Selmer J., Lauring J. (2016). Work engagement and intercultural adjustment. Int. J. Cross Cult. Manag..

[82] Kim W., Park J. (2017). Examining structural relationships between work engagement, organizational procedural justice, knowledge sharing, and innovative work behavior for sustainable organizations. Sustainability.

[83] Llorens S., Schaufeli W., Bakker A., Salanova M. (2007). Does a positive gain spiral of resources, efficacy beliefs and engagement exist?. Comput. Hum. Behav..

[84] Chahal H., Bakshi P. (2015). Examining intellectual capital and competitive advantage relationship: Role of innovation and organizational learning. Int. J. Bank Mark..

[85] Wang C.-J., Tsai C.-Y. (2014). Managing innovation and creativity in organizations: An empirical study of service industries in Taiwan. Serv. Bus..

[86] Kör B., Wakkee I., van der Sijde P. (2021). How to promote managers’ innovative behavior at work: Individual factors and perceptions. Technovation.

[87] Brislin R.W. (1980). Translation and content analysis of oral and written materials. Handbook of Cross-Cultural Psychology.

[88] Den Hartog D.N. (2015). Ethical leadership. Annu. Rev. Organ. Psychol. Organ. Behav..

[89] Albrecht S.L. (2012). The influence of job, team and organizational level resources on employee well-being, engagement, commitment and extra-role performance: Test of a model. Int. J. Manpow..

[90] Yalabik Z.Y., Popaitoon P., Chowne J.A., Rayton B.A. (2013). Work engagement as a mediator between employee attitudes and outcomes. Int. J. Hum. Resour. Manag..

[91] Chen G., Gully S.M., Eden D. (2001). Validation of a new general self-efficacy scale. Organ. Res. Methods.

[92] Azizli N., Atkinson B.E., Baughman H.M., Giammarco E.A. (2015). Relationships between general self-efficacy, planning for the future, and life satisfaction. Personal. Individ. Differ..

[93] Crane M.F., Brabazon G., Gucciardi D.F., Loveday T., Wiggins M. (2017). General self-efficacy and psychological resilience promote skill acquisition rate under psychological pressure. Australas. J. Organ. Psychol..

[94] Javed B., Khan A.K., Arjoon S., Mashkoor M., Haque A.U. (2020). Openness to experience, ethical leadership, and innovative work behavior. J. Creat. Behav..

[95] Liang H., Saraf N., Hu Q., Xue Y. (2007). Assimilation of enterprise systems: The effect of institutional pressures and the mediating role of top management. MIS Q..

[96] Podsakoff P.M., MacKenzie S.B., Lee J.-Y., Podsakoff N.P. (2003). Common method biases in behavioral research: A critical review of the literature and recommended remedies. J. Appl. Psychol..

[97] Li Y., Liu H., Lim E.T., Goh J.M., Yang F., Lee M.K. (2018). Customer’s reaction to cross-channel integration in omnichannel retailing: The mediating roles of retailer uncertainty, identity attractiveness, and switching costs. Decis. Support Syst..

[98] Hammond M.M., Neff N.L., Farr J.L., Schwall A.R., Zhao X. (2011). Predictors of individual-level innovation at work: A meta-analysis. Psychol. Aesthet. Creat. Arts.

[99] Atinc G., Simmering M.J., Kroll M.J. (2012). Control variable use and reporting in macro and micro management research. Organ. Res. Methods.

[100] Hayes A. (2012). PROCESS: A versatile computational tool for observed variable mediation, moderation, and conditional process modeling. http://www.afhayes.com/public/process2012.pdf.

[101] James L.R., Demaree R.G., Wolf G. (1984). Estimating within-group interrater reliability with and without response bias. J. Appl. Psychol..

[102] LeBreton J.M., Senter J.L. (2008). Answers to 20 questions about interrater reliability and interrater agreement. Organ. Res. Methods.

[103] Kozlowski S.W., Klein K.J. (2000). A multilevel approach to theory and research in organizations: Contextual, temporal, and emergent processes. Multilevel Theory, Research, and Methods in Organization: Foundations, Extensions, and New Directions.

[104] Bliese P.D. (1998). Group size, ICC values, and group-level correlations: A simulation. Organ. Res. Methods.

[105] Gerbing D.W., Anderson J.C. (1988). An updated paradigm for scale development incorporating unidimensionality and its assessment. J. Mark. Res..

[106] Fornell C., Larcker D.F. (1981). Evaluating structural equation models with unobservable variables and measurement error. J. Mark. Res..

[107] Hair J., Anderson R., Tatham R., Black W. (1998). Multivariate Data Analysis.

[108] Hair J.F., Sarstedt M., Hopkins L., Kuppelwieser V.G. (2014). Partial least squares structural equation modeling (PLS-SEM): An emerging tool in business research. Eur. Bus. Rev..

[109] Henseler J., Ringle C.M., Sarstedt M. (2015). A new criterion for assessing discriminant validity in variance-based structural equation modeling. J. Acad. Mark. Sci..

[110] Dohoo I.R., Ducrot C., Fourichon C., Donald A., Hurnik D. (1997). An overview of techniques for dealing with large numbers of independent variables in epidemiologic studies. Prev. Vet. Med..

[111] Haines-Gadd L. (2015). Does TRIZ change people? Evaluating the impact of TRIZ training within an organisation: Implications for theory and practice. Procedia Eng..

[112] Lisbona A., Palaci F., Salanova M., Frese M. (2018). The effects of work engagement and self-efficacy on personal initiative and performance. Psicothema.

[113] Nazir O., Islam J.U. (2020). Influence of CSR-specific activities on work engagement and employees’ innovative work behaviour: An empirical investigation. Curr. Issues Tour..

[114] Khan M.M., Mubarik M.S., Islam T. (2020). Leading the innovation: Role of trust and job crafting as sequential mediators relating servant leadership and innovative work behavior. Eur. J. Innov. Manag..

[115] Khan M.M., Mubarik M.S., Islam T., Rehman A., Ahmed S.S., Khan E., Sohail F. (2021). How servant leadership triggers innovative work behavior: Exploring the sequential mediating role of psychological empowerment and job crafting. Eur. J. Innov. Manag..

[116] Ashfaq F., Abid G., Ilyas S., Hasnain A. (2021). How transformational leadership influences innovative behavior: The mediating role of psychological empowerment and proactivity of employees. Indep. J. Manag. Prod..

[117] Patthamasukhon I. (2022). The Next Asian Digital Behemoth: Thailand’s 4.0 Revolution. https://www.thailandnow.in.th/business-investment/the-next-asian-digital-behemoth-thailands-4-0-revolution/.

